# Transition from paediatric to adult care of adolescents living with HIV in sub-Saharan Africa: challenges, youth-friendly models, and outcomes

**DOI:** 10.7448/IAS.20.4.21528

**Published:** 2017-05-16

**Authors:** Désiré Lucien Dahourou, Chloé Gautier-Lafaye, Chloe A. Teasdale, Lorna Renner, Marcel Yotebieng, Sophie Desmonde, Samuel Ayaya, Mary-Ann Davies, Valériane Leroy

**Affiliations:** ^a^ Centre of International Research for Health, Faculty of Health Sciences, University of Ouagadougou, Ouagadougou, Burkina Faso; ^b^ Clinical Research Department, Centre Muraz, Bobo-Dioulasso, Burkina Faso; ^c^ Centre Hospitalier Universitaire de Toulouse, Toulouse, France; ^d^ ICAP, Mailman School of Public Health, Columbia University, New York, NY, USA; ^e^ Korlebu Hospital, Accra, Ghana; ^f^ Division of Epidemiology, The Ohio State University, College of Public Health, Columbus, OH, USA; ^g^ Inserm U1219, Bordeaux University, Bordeaux, France; ^h^ Moi Hospital, El-doret, Kenya; ^i^ Centre for Infectious Disease Epidemiology and Research, School of Public Health and Family Medicine, University of Cape Town, Cape Town, South Africa; ^j^ Inserm, Laboratoire d’Epidémiologie et Analyses en Santé Publique (LEASP) - UMR 1027, Université Toulouse 3, Toulouse, France

**Keywords:** HIV infections, adolescents, youth, transition, Africa

## Abstract

**Introduction:** The number of adolescents with perinatally or behaviourally acquired HIV is increasing in low-income countries, and especially in sub-Saharan Africa where HIV prevalence and incidence are the highest. As they survive into adulthood in the era of antiretroviral therapy, there is a pressing need to transfer them from paediatric to adult care, known as the transition of care. We conducted a narrative review of recent evidence on their transition outcomes in Africa, highlighting the specific needs and challenges in these populations and settings, and the different models of care for transition.

**Areas covered:** We searched PubMed bibliographic database, HIV conference content, and grey literature from January 2000 to August 2016 with the following keywords: HIV infections AND (adolescents or youth) AND transition AND Africa. All qualitative and quantitative, experimental and observational studies including HIV-infected patients aged 10–24 years with information on transition were eligible.

**Results:** Few data on transition outcomes for HIV-infected adolescents are available from Africa settings. Studies mainly from Southern and East Africa reported on the barriers to successful transition, highlighting several gaps. These included lack of adequate infrastructure, staff training and communication between paediatric and adult clinicians as well as the fear of stigma of adolescents and youth living with HIV. Most countries have no specific national guidelines on when to disclose HIV status or when and how to transition to adult care. Several models of care adapted to the adolescent transition question have been implemented in specific settings. These models include teen clinics, peer educators or the use of social media. However, regardless of the model, services are increasingly overburdened and have insufficient human resources. Furthermore, very high attrition has been observed among adolescents and youth compared to younger children or older adults. There is a need to identify sub-groups at higher risk of loss to follow-up for targeted care and peer support.

**Expert commentary:** Although the available HIV-related data on adolescent transition outcomes are limited, there is evidence of their increased vulnerability during this period. Standardized data gathering, analysis, and reporting systems specific to adolescent transition are essential to improve understanding and adolescent outcomes in Africa.

## Introduction

Perinatally or behaviourally HIV-infected adolescents (10–19 years) and young adults (20–24 years) are an increasing proportion of the HIV-infected population. In 2014, according to UNICEF, an estimated 2 million adolescents were living with HIV worldwide, with 25% living in only three African countries (South Africa, Nigeria, and Kenya) [1]. In addition, 2.8 million youth aged 20–24 years were living with HIV in 2014; of these, about 82% lived in sub-Saharan Africa and the majority (71%) were females [2,3]. Incidence of HIV remains alarmingly high in adolescents and youth aged 10–24 years in Africa. According to UNAIDS, 37% of new HIV infections occurring in sub-Saharan African adults in 2015 were among adolescents and youth aged 15–24 years, especially among women; this age group represented 34% of the adult population living with HIV in 2015 [3].

The rollout of antiretroviral therapy (ART) has allowed perinatally HIV-infected children to survive into adulthood, but this growing population faces emerging challenges. A key issue of concern is the transition of adolescent patients from paediatric to adult-centred care settings. This process, known as transition of care, involves more than changing location of services from the paediatric to the adult care clinics. It also involves adolescents living with HIV taking responsibility for their own health and disease management, raising new challenges for sustaining retention in the care continuum.

Indeed, adolescents and youth living with HIV in Africa represent a vulnerable population who face developmental, psychosocial and comorbidity issues while coping with a chronic, potentially stigmatizing disease [4,5]. They struggle with more health-related issues than their uninfected counterparts: clinical problems due to the long-term exposure to the virus and ART including drug toxicity, metabolic and cardiovascular disorders, chronic lung disease, renal and bone diseases and neurocognitive disorders [4]. Growth and puberty are commonly delayed and they face psychological challenges, desiring acceptance by their peers, while trying to achieve self-assertion and autonomy over their disease [5]. These latter issues may be more pertinent for perinatally infected adolescents compared to behaviourally infected adolescents. Adolescents and youth living with HIV are also at high risk of depression [5,6], may deny or be unaware of their HIV status [7], and as adolescents are prone to risk taking [8]. This vulnerable period during adolescence can lead to higher risks of HIV transmission to uninfected sexual partners, treatment interruption and overall adherence problems, treatment failure and loss to follow-up from HIV care. Data show that while ART access has greatly improved survival among people living with HIV and AIDS-related deaths have declined overall, adolescents are the exception and AIDS-related illnesses have been reported to be the leading cause of death among adolescents in Africa [9].

The success of the transition process from paediatric to adult healthcare settings will greatly impact the success of ART in adolescents and young adults [10]. The transition of care involves complex changes of clinics, models of care, and healthcare providers. For adolescents living with HIV, these changes can lead to care disruptions occurring during a vulnerable period. If this transition is not well conducted, there is a high risk of non-adherence to ART, emergence of viral resistance and loss to follow-up, with implications for individual patients as well as for the overall epidemic.

The 90–90–90 treatment targets were developed in 2014 by UNAIDS with the aim to achieve 90% HIV testing coverage, 90% ART coverage among those diagnosed with HIV, and 90% viral suppression among those on ART [11,12]. The effectiveness of integrating implementation science research efforts with current policies, guidelines and recommendations for effective transitioning process needs to be assessed, including the evaluation of the 90–90–90 targets outcomes post-transition [10].

Adolescent transition outcomes have been reported elsewhere in high- and middle-income countries in North America, Europe, and Asia [13–21], but these types of assessments are less available in sub-Saharan Africa, because the limited-resource context increases the complexity of transition and its evaluation. In Sub-Saharan Africa, the healthcare settings in which adolescents may be receiving care are characterized by the lack of specialized providers, specifically a dearth in adolescent medicine expertise, and overburdened clinics characterized by crowded spaces [22]. We conducted a narrative review of the recent evidence of specific needs and challenges of adolescents and young adults’ transition in sub-Saharan African populations, highlighting the different existing models of care for transition and transition outcomes.

## Methods

We searched the PubMed bibliographic database from January 2000 to August 2016 as well as content from the 2014–2016 International AIDS Society conferences, the Conference on Retroviruses and Opportunistic Infections, and International Workshop on HIV Pediatrics using the following keywords: HIV infections AND (adolescents or youth) AND transition AND Africa. All surveys, experimental and observational studies with quantitative or qualitative data on HIV-infected patients aged 10–24 years with transition information were eligible. The final reference list, which included peer-reviewed published papers, scientific and technical reports as well as conferences abstracts, was hand screened, and reviewed (VL, DD, CGL, CT).

We extracted all qualitative and quantitative data highlighting expectations, reported needs and specific challenges associated with adolescent transition in these settings from both healthcare providers and adolescents and youth living with HIV, along with descriptions of specific youth-friendly transition models of care. In general, adolescents are defined as ages 10–19 while youth are defined as ages 20–24. We also report evidence of post-transition outcomes (adherence, treatment outcomes, retention in care and survival), focusing on available data from several large cohorts, including the IeDEA collaboration [23], Optimal Models (ICAP-Columbia University) [24], Medecins sans Frontieres [25,26] and Baylor University International Pediatric AIDS Initiative (BIPAI) [27,28], as well as national programme data.

## Results

Among the 64 articles and 17 conference abstracts identified through database searching, 53 and 10, respectively, were excluded because they were not directly related to adolescent transition in HIV care. Overall, 20 studies were selected for data extraction: 11 full-text articles, 7 conference abstracts and two unpublished studies. There were a few studies over multiple sub-Saharan African countries, but most were conducted in South Africa or East Africa (Uganda, Kenya).

## Barriers, facilitators, expectations and needs associated with transition: the provider’s and adolescent’s points of view

Nine studies (4 articles; 5 abstracts, all but one conducted in East and Southern Africa) including quantitative surveys and qualitative research studies were conducted with both healthcare providers and young adult populations, to better understand the challenges, expectations, and needs related to transition (Table 1). Despite the increase in the number of adolescents and young adults presenting to care, most clinics’ services did not take into account the specific needs of this growing population. Overall, several gaps were consistently highlighted in transition services.

**Table 1.** Quantitative and qualitative studies on the barriers, expectations and needs about youth transition services in sub-Saharan Africa: the youth and provider points of view

Reference, Year	Site	Study Design	Population	Results
Soeters, AIDS 2014, MOPE083 [29]	Burundi, Ethiopia, Kenya, Rwanda, Tanzania and Uganda	Cross-sectional survey of adolescent care providers’ clinicians about the gaps in their transition services	81 HIV practitioners from 20 health facilities in Burundi, Ethiopia, Kenya, Rwanda, Tanzania and Uganda	Almost half of facilities did not implement transition plans and a similar proportion of practitioners were not trained to support transition. The most frequent transition age was 18 years (range: 13–22 years). Operational obstacles: lack of specialist adolescent-friendly services, training, policy, guidelines and planning. Healthcare providers recommended service improvement in patient tracking, early preparation for transition, psychosocial support and health education
Pettit, African journal of reproductive Health, 2013 [22]	Sub-Saharan Africa	Multicountry assessment (Botswana, Uganda, Tanzania, Mozambique, Malawi, Zimbabwe, Kenya, Rwanda, South Africa, Swaziland)	34 semi-structured interviews: 26 key informants (ministries of health, HIV service providers of adolescents living with HIV) and 8 young adult peer educators, aged 18–25, between February and April 2011	Both providers and peer educators agreed on the major themes, gaps and recommendations: 1) Drug access and availability: Access to ART, and third line, is a challenge. 2) Clinical care: Adolescent-friendly services and staff training are needed. Adherence is a huge challenge. 3) Nutritional care: malnutrition is frequent and requires clinic support. 5) Sexual and Reproductive Health: fear of rejection, stigma, and blame induce of lack of disclosure to sexual partners with a risk of sexual transmission. Lack of sexual reproductive health training. 6) Psychological support: Health providers have insufficient training in mental health and disclosure. Peer support is common 7) Social support: some actions to help for transport are needed. 8) Transition care is a challenge: many adolescents and also paediatric providers may be resistant to transition. 9) Resources: Policymakers need to develop clinical guidelines for healthcare transition.
Kung, S Afr Med J, 2016 [30]	Western Cape Province, South Africa	Interviews and self-administered survey on the current state of the transition, barriers and facilitators, and model components	Seven physicians and counsellors in adolescent/paediatric care, from five clinics, were interviewed, and 43 completed a written survey	One barrier identified was the healthcare providers’ difficulty in letting go of their relationships with the adolescent patients. Healthcare providers felt a strong and protective attachment towards them. A second barrier identified was a lack of structure and effective communication between adult and paediatric providers; healthcare providers feared that they were transferring their adolescents unprepared, to a judgmental, depersonalized and overburdened environment. All interviewees and a majority of survey respondents (80%) agreed that the formation of adolescent support groups in adult care clinics as well as a later transition age would improve the transition process
Snyder, AIDS 2014, MOPE084 [31]	Western Cape, South Africa	Quantitative and qualitative mixed-methods study to document barriers to transitioning and the perceived utility and feasibility of specific interventions	92 adolescents pre- and post-transition (15–25 years) and 43 healthcare providers completed survey and interviews	The most salient, perceived barriers to transitioning include adolescents’ mental health difficulties, provider relationship disruption, and stigma. In addition to losing solidarity with other adolescents and entering a physically and socially less comfortable space, adolescents fear judgment from adult patients in crowded clinic waiting areas. Written guidelines, strengthened communication channels, and support groups were deemed most feasible and potentially effective, and were strongly recommended. A settings-based approach to improving the atmosphere for transitioning adolescents is also needed
Ouedraogo, Global Health promotion, 2012 [32]	Ouagadougou, Burkina Faso	Qualitative study from 2006 to 2008 to document the own perception of HIV-infected young women on the challenges of reaching adulthood	21 HIV-infected single women, 16–28 years	Becoming aware of seropositive status produces a biographical disruption that introduce a “before” and “after”, it erodes the image of their self which is still in construction and transform their transition phase to adulthood (question of marriage and procreation which are the keystone). These young women adopt strategies to overcome these vulnerabilities by concealing their HIV status to reconstruct the “self”, to their partner. The strategies differ from one person to another, with the same goal: social success. So, exposing their sexual partner to the risk of transmission puts them in a legally and morally vulnerable position
Siu, Aids Care, 2012 [33]	Kampala, Uganda	Qualitative study using in-depth interviews and focus group to describe HIV disclosure practices and concerns from the perspective of young adults	Transition clinic of the Infectious diseases Institute: 20 young adults (10 males and 10 females) both vertically and horizontally infected aged range 15–23	Disclosure was perceived as a broad concept that goes beyond the act of telling other about one’s serostatus. Joking to “test the water” and emotionally prepare the disclosure before later disclosing more seriously was a disclosure strategy described by young adults. They felt their secret was frequently violated by their status disclosure to a third party.
Chekata Inzaule C, AIDS 2016 [34]	Uganda	Qualitative analysis using in depth interviews and focus groups	Sample of 24 interviews and 2 focus groups	Adolescents reported lack of family and clinical support as barriers, also cited transition from paediatric to adult care and declining peer to peer support; treatment holidays, perceived discrimination and stigma at boarding school also reported
Massavon W, AIDS 2016 [35]	Kampala, Uganda	Qualitative study to examine factors, barriers and challenges to transfer from the adolescents’ clinic to the adult ART clinic at one health facility. Semi-structured interviews.	132 youth, 17–28 years on ART, data collected over 12 months, period 2014–2015	Adolescent clinic specified but without description of model of care; model of transition called “Transition Care Counselling” to prepare adolescents for adult care. Mean age 20.1 years, 96% on ART, 65% accepted transfer to adult clinic but only 12% transferred within 12 months, reported enabling factors included perceptions of maturity, financial security and other support (feeling safe and secure in clinic); barriers and challenges included financial insecurity, no support, user fees, stigma from adults and unfavourable adult clinic appointments, breaking-up of peer support networks and emotional and psychological unpreparedness
Gillespie N, AIDS 2016 [28]	Gaborone, Botswana; Maseru, Lesotho; Lilongwe, Malawi; Mbabane, Swaziland; Mbeya, Tanzania; Mwanza, Tanzania; and Houston, Texas, USA	Description of characteristics and current healthcare transition practices across Baylor International Paediatric AIDS Initiative	3,060 adolescents 15–19 years and 2797 youth ≥20-years (upper age not specified) enrolled in care at 7 Baylor International Paediatric AIDS Initiative sites, almost all presumed perinatally infected	263 patients transferred from BIPAI clinics in 12 months; healthcare worker respondents cited concerns regarding transition readiness of patients and lack of support services outside of paediatric clinic as potential barriers

First, there was a lack of infrastructure, and few programmes reported having dedicated structured transition space in the clinic with adult care often delivered in depersonalized and overburdened environments [30]. Second, most of adolescent HIV care providers in sub-Saharan Africa have acknowledged gaps with regards to understanding and managing transition as a separate process, especially those related to clinical capacity and guidelines, including training for healthcare workers and national and institutional policies [29]. The clinical, psychological, social, and reproductive health needs of adolescents and young adults remain poorly understood by practitioners and efforts to mobilize and advocate for their treatment, care, and support have been inadequate until recently. Practitioners also reported that adolescents and young adults were generally unprepared clinically and psychologically for their transition into adult care [29], with paediatric healthcare providers feeling a strong and protective attachment towards these patients. Third, there has been a lack of communication between adolescent and adult care physicians around transition. Finally, adolescents and young adults feel stigmatized and fear disclosure of their HIV status in HIV adult clinics [30].

Major gaps in the area of peer support for adolescents and youth were also consistently reported in adult care settings by both care providers and peer educators [22]. Adolescents and young adults had specific expectations of transition services; as reported in Burkina Faso, reaching adulthood raises particular problems for HIV-infected adolescent females with the issues of relationship to self (biographical disruption and jeopardized female identity) and to others (weakening of social ties in situations of dependency and of pursuit of social advancement, marriage and procreation) [32]. In one study, female adolescents and youth engagement in the prevention of mother-to-child HIV transmission (PMTCT) services showed that they had poorer antenatal care attendance and uptake of ART compared to adult pregnant women [36]. This vulnerability needs to be taken into account when managing the transition process [32]. More generally, perceived stigma, and lack of support are concerns expressed by both male and female adolescents and young adults [31–35].

Key recommendations to tackle these barriers included the need for better planning and preparation for clinical providers and adolescents to improve the transition process, with a focus on improving both clinical and psychosocial support throughout the process [29]. For instance, the use of multidisciplinary staff was recommended. As in South Africa, the need for a systematic routine preparation of adolescents before their transition was highlighted [30]. Suggestions for this preparation included meetings with the care providers, parents and also peers to improve communication. In a qualitative multicountry assessment of youth key informants (staff and peer educators) on the needs of adolescents and youth in 10 sub-Saharan African countries, it was reported that comprehensive, adolescent-friendly services that champion both peer support and collaboration between healthcare service providers can promote successful transitions into adulthood [22] (Table 1). Another suggestion was to improve the atmosphere for transitioning adolescents to adult care by creating a dedicated space in the clinics for transitioning patients [29,30]. Where dedicated space was not feasible, dedicated visit days were suggested for adolescents and youth. Although not representative of all sub-Saharan Africa, these studies consistently suggest a model of care which supports the psychosocial needs and logistics of transitioning adolescent patients into adult care without overburdening the health systems [31]. Finally, it was felt crucial to offer to adolescents a holistic package of care approach to meet their clinical, nutritional, psychosocial, sexual, PMTCT needs over their transition process, as recommended by Petit et al. [22] (Table 1).

## Implementation of youth-friendly models of care for transition in sub-Saharan Africa

Despite extensive evidence on the need for adolescent and youth-friendly services, particularly to facilitate transition, such services are rarely implemented in African settings. The adolescent transition could occur through different settings and models of care currently practiced in different African contexts; however, it is often the case that healthcare centres are overbooked with care occurring in crowded spaces, with too few healthcare staff who are not trained to work with adolescents and youth [22,30].

When transition occurs is a crucial consideration in the African setting. Most of the paediatric clinical services follow children only until age 15 after which patients are expected to attend adult services, but this may be different with chronic diseases such as HIV, with both paediatricians and adolescents tending to delay the transition time [37,38]. We did not find any national guidelines specifying an age for transition; however, in practice, we observed from available data that transition tends to occur across a wide age range (e.g. 13–22 years), with most occurring after 18 years [29], 20 years [35,39], or 22 years [40]. While chronological age is a standard criterion, it is also important to consider the age of maturity of the patient; in other disciplines, providers suggest that age of maturity may vary depending on social support, education, and learning capabilities [38].

In our review, we identified four models of transition for adolescents. The standard model most often used in resource-limited settings was the transition of adolescents from paediatric care clinics to a distinct adult care clinic as decided by the providers. In the second model, the same providers cared for both children and adults in the absence of a clearly defined family-centred approach. Another model of care was for adolescents to remain within the same clinic with fully integrated family-centred approaches to care, usually developed and assessed within the implementation of prevention of mother-to-child intervention programmes [41,42]. The fourth model of care was youth-friendly transition clinics which have been developed in several settings to specifically help adolescents and youth manage on their own HIV disease, support adherence to ART and retention in follow-up through dedicated care prior to moving the adolescent to an adult clinic.

A review of national programmes and approaches to adolescent disclosure of HIV status and transition to adult HIV care among country programmes participating in the *New Horizons Advancing Pediatric HIV Care* collaboration was conducted in 2015 [43]. Data were collected from four national HIV/AIDS programmes (Kenya, Zambia, Swaziland, and Lesotho). All four countries supported discussions about disclosure with the naming of HIV at ages 4–8 years, full disclosure was supported in three countries by age 10 years and all countries required full disclosure before transition to adult care. While all four countries reported national health strategies focusing on adolescents in guidelines, only Kenya has a national adolescent package of care including standardized national training and monitoring tools for transition to adult care. There were no national guidelines in any country indicating an age for transition, but the reported standard practice in these countries is 15 years and older.

Different youth-friendly models of care have been implemented; however, very few have been described and assessed in Africa (Table 2). The Botswana-Baylor Children’s Clinical Centre of Excellence has implemented a comprehensive healthcare transition programme, using locally adapted tools to assist adolescents and youth in achieving a successful transition into adult care [44]. The Baylor “Kalogo” Transition Programme model is conducted by a facility-based multidisciplinary transition team which includes peer educator adolescents. The programme uses five tools: a transition roadmap to assess the adolescent’s knowledge, skills and adherence; a risk-screening tool to identify risks for adherence/treatment failure prior to transition; a tool for providers to ask two to three transition readiness questions at each visit; homework assignments to reinforce education given at clinic visits; and an educational module on transition during peer support group meetings. In 2016, among 1000 adolescents aged 13–19 years enrolled in the programme, only 33 (<3%) were identified as ready for transition in peer groups. The Baylor programme has also been implemented in Malawi with transition training aimed to build economic, psychosocial and self-care skills to help prepare adolescents and youth to a successfully transition into adulthood, and to become mentors themselves [39] (Table 2).

**Table 2.** Assessment of youth-friendly models of care: ongoing research in sub-Saharan Africa

Reference, Year	Site	Study Design	Population	Models of care	Outcomes
Patten, J Int AIDS Soc 2013 [45]	Khayelitsha, South Africa	Retrospective before-and-after observational cohort with data collected from May 2010 to April 2011 when CD4+ count are tested in laboratory (Group A) and from August 2011 to July 2012 with same day point-of-care (POC) CD4 testing (Group B) to assess whether there was an associated reduction in attrition between HIV testing, and ART initiation	576 adolescents and young adults living with HIV, ART-naïve and probably recently diagnosed, 12–25 years, 272 in group A and 304 in group B	Youth clinic and offer youth-friendly services to address the needs of this difficult population group. June 2011 POC CD4 cell-count testing was introduced in youth clinic. Both had 3 ART preparation counselling session	– Group B more receive CD4 cell count test result and their eligibility assessed (90% vs. 67%; relative risk [RR] = 2.4, 95%CI:1.8–3.4, *p* < 0.0001) – No significant difference in the proportion starting and completing ART preparation counselling sessions 56% vs. 58% (*p* = 0.9). – 8 days reduction in the time from HIV testing to ART initiation in Group B, (*p* = 0.6). – The proportion of eligible patient who initiated ART was 44% and 50% (*p* = 0.6) in group A and group B, respectively, and a similar proportion were retained on therapy at three months after initiation (RR = 1.0, 95% CI:0.5–1.2, *p* = 0.9) – No difference in the proportion of patients lost to follow-up
Nyabigambo, Adolescent health, Medicine and Therapeutics 2014, AIDS care 2014 [40,46]	Kampala, Uganda	Cross-sectional design and quantitative methods to collect data to study the levels (regular/irregular) and determinants (personal, health service delivery and community) of HIV transition clinic (HTC) services utilization by adolescents and young adults living with HIV	379 adolescents and young adults 15–24 years, registered clients at an HTC between March and June 2012	Infectious disease institute, with Wednesday monthly visits, and providing clinical examination, laboratory services, prevention mother-to-child transmission services, family planning services, treatment of sexually transmitted infections, ART psychosocial support, counselling, home visiting, peer support services, skills building programmes	– 32% were regular utilizers of the HTC, mean age 22 years, 61% currently on ART. – 82% of regular utilizers were females No relationship between reported wellbeing (measured with General Well-Being Schedule, 18-point scale) and attending all clinical visits (compared to missing at least one visit) – The most utilized services were: clinical examination (96%), laboratory (87%) and counselling (70%), – The less utilized: home visiting (6%,) peer support (20%). Individual correlates of HTC utilization – urban location: regular 56% vs. irregular 69%, *p* = 0.016 – age 15–19: regular 15% vs. irregular 9%, *p* = 0.044 – currently on ART: regular 82% vs. irregular 51%, *p* = 0.000 – last CD4 < 250: regular 37% vs. irregular 18%, *p* = 0.000 Community correlates: – not having a caregiver at home: regular 11% vs. irregular 22%, *p* = 0.014 Health services delivery correlates – no receiving counselling: regular 20% vs. irregular 36%, *p* = 0.001 Multivariable analysis: CD4 > 251 (adjusted Odds Ratio [AOR] = 0.58 95% CI = 0.36–0.95), not being on ART (AOR = 0.47, 95% CI = 0.15–0.47), not receiving counselling (AOR = 0.47, 95% CI = 0.27–0.83)
McKenney, 2016 unpublished [39]	Lilongwe, Malawi	Assessment of a Transition Training programme, in Baylor College teen Clubs	800 adolescents, 18–24 years, 106 graduate participants, from 2013 to 2015	6-week Transition Training programme to transfer to adolescents economic, psychosocial, and self-care skills needs to make a successful transition into adulthood	Mean age: 20 years 23% have disclosed their HIV status to friends/partners; 25% were enrolled in secondary school, and 3% in university; 10% found employment, 8% were involved as ambassadors for adolescents and young adults; 22% were mentors or peers for teen clubs
Henwood, Aids Care, 2016 [25]	Khayelitsha (Cape Town), South Africa	Self-administered survey and focus groups of MSF youth club members using virtual chat support room	60 adolescents and young adults enrolled in MSF youth clubs surveyed; 12 in focus group	MSF youth care for 12-25-year olds includes “youth clubs” which include “MXit” a cell-phone based virtual chat room for social networking and support	58% of survey respondents were 23-25 years and 83% had a cell phone. 60% had used MXit. 84% felt that offering a service outside the youth club meetings was important; cost and anonymity were concerns

In Khayelisha, South Africa, a differentiated model of care for youth has been piloted by Medecins Sans Frontieres (MSF) starting in 2011, offering three innovative interventions aimed at reducing key gaps in the HIV cascade (Table 2): (1) same-day point-of-care (POC) CD4 testing, targeting losses between HIV diagnosis and ART eligibility; (2) rapid ART initiation with supportive counselling; (3) youth-clubs targeting losses from HIV diagnosis through the continuum of care [26]. POC CD4 testing was shown to significantly reduce attrition between HIV-testing and assessment of ART eligibility [45] (Table 2). However, additional strategies to improve uptake of ART are needed, for instance improving patient support for HIV-positive youth immediately after diagnosis. In order to support youth on ART, the MSF youth clubs include “MXit” a cell-phone-based virtual chat room for social networking and support [25]. Reported usage of the MXit chat room was only 60% among a sample of 90 club members who participated in an evaluation conducted by MSF, but participants indicated acceptance of the platform and a desire to interact with their peers through social media. Suggestions from youth about measures to improve the platform included accessible chat histories, using more interfaces such as Facebook or WhatsApp, and to have topical discussions containing pertinent information for youth.

In Kampala, Uganda, the “HIV Transition Clinic” (HTC) a youth-friendly transition clinic, was implemented offering specific HIV services one day a week dedicated to adolescents and youth (Table 2). However, regular utilization of these services by adolescents and youth living with HIV was found to be low, reaching only 32% in 2012. In an analysis of factors associated with utilization, it seemed to be HIV infection stage and availability of HIV counselling services were associated with greater attendance while socio-demographic or community factors were not [40,46]. Further studies of this model are needed to measure post-transition outcomes among adolescents and youth accessing HTC care.

Whatever the model of care for transition, sub-Saharan HIV-care services are increasingly overbooked, with few human resources [22]. This increases considerably the challenges to offer appropriate, individualized, and durable HIV-care services to adolescents and young adults.

## Adolescent and youth post-transition outcomes in sub-Saharan Africa

Data on adolescents and youth suggest that they have poorer retention both before and after ART initiation compared to both paediatric and older adult populations. In a large cohort from 160 HIV care and treatment facilities in Kenya, Mozambique, Tanzania and Rwanda, patients aged 15–24 were 50% more likely to be classified as lost to follow-up (LTFU) prior to ART compared to 25–54-year olds (adjusted hazards ratio 1.50, 95% CI 1.45–1.54) and almost 60% more likely to be LTFU after ART initiation (adjusted hazards ratio 1.59 (1.52–1.67) [47]. It is possible that the high rate of LTFU among adolescents and youth is associated with transition from paediatric to adult care. Few studies have explored the impact of transition on retention among adolescents and youth in resources-limited settings or examined interventions aimed at addressing transition challenges (Table 3). In an analysis from Khayelisha, although there was no comparison group, the overall 12-month retention was high among adolescents and young adults attending youth clubs, reaching 82%, and it was significantly higher among those who were stable on ART [26]. In South Africa, adolescents attending a dedicated Saturday Teen clinic had higher retention in care and viral suppression rates compared to adolescents attending a standard paediatric clinic (Table 3).

**Table 3.** Adolescent transition outcomes in sub-Saharan Africa

Reference, Year	Site	Study Design	Population	Models of care	Outcomes
Lamb, AIDS 2014 [47]	Kenya, Mozambique, Tanzania, Rwanda	Retrospective cohort comparing pre and post ART attrition (=LTFU or death 1 year after enrolment or ART initiation) rates between adolescents and young adults (15–24) and other patients. Patient-level and clinic-level factors associated with attrition were similarly assessed among youth with multivariate models	312,335 ≥ 10 years of age enrolling in HIV care between 01/05 and 09/10 at 160 clinics	Transition from a paediatric to distinct adult sites. All sites receiving financial support from ICAP at Columbia University through PEPFAR funding	– pre-ART attrition rates were higher among adolescents and young adults compared with other groups. – post- ART initiation rates were higher in adolescents and young adults compared with other groups – Adolescents and young adults attending clinics providing sexual and reproductive health services including condoms (adjusted Hazard Ratio [AHR] = 0.47 95%CI: 0.32–0.70) and clinics offering adolescent support group (AHR = 0.73 95% CI: 0. 52–1.0) experienced significantly lower attrition after ART initiation. – Adolescents and youth enrolled with CD4 cell count lower than 100/mL or higher than 200/mL had a significant higher pre-ART attrition than those between 100 and 200 cells/mL. Those with missing CD4 at ART initiation, CD4 >350 and those <100/mL had higher attrition compared with those initiating ART between 100 and 350 cells/mL. – enrolling into HIV care while on treatment for TB lower attrition in pre-ART phase (RR = 0, 60, 95%CI: 0, 48–0, 73) but no difference in attrition after ART initiation. – Kenya, Mozambique, Tanzania had higher pre-ART attrition compared with adolescents and young adults attending clinics in Rwanda
MSF report, 2011–2015, unpublished [26]	Khayelitsha, Cape Town, South Africa	Prospective cohort providing a youth differentiated model of care: with same day point of care CD4 testing and rapid ART initiation, without comparison group	337 HIV-infected adolescents and young adults, 12–25 years, enrolled from 2012 to 2015	MSF-funded youth clubs with psychosocial support, HIV clinical management, family planning, linkage to mentor via mobile phone	Overall 12-month retention outcome: 82% (95% CI: 76–86%). Varying significantly by enrolment category (*p* < 0.001): Ineligible to start ART: 53% (95% CI: 40–64%) Newly initiated on ART: 86% (95% CI: 79–91%) Stable on ART: 94% (95% CI: 85–97%)
Zanoni, (Int HIV ped W 2016) [48]	KwaZulu-Natal, South Africa	Retrospective cohort comparing a Saturday teen clinic compared to standard weekday paediatric clinics	254 perinatally HIV-infected adolescents	Saturday teen clinic was implemented was dedicated peer support and structured social activities after 6 months on ART	Overall viral suppression was 85% and retention 89%. Significantly higher retention rates in adolescents attending the dedicated teen clinic (97%) versus adolescents in standard care (85%) (*p* = 0.0005)
Teasdale, C. J.Aquir Immune Defic Syndr 2016 [49]	Nyanza, Kenya	Retrospective analysis comparing LTFU pre-YAFS (youth- adolescent-friendly services) to LTFU post YAFS in 6 YAFS. In addition LTFU outcomes were examined in the pre (03–12/2011) and post YAFS periods (312/2013) at 28 health facilities that did not implement YAFS to examine changes in LTFU in the same periods which were unrelated to YAFS. The analysis examined LTFU before ART and LTFU after ART	2321 HIV-infected adolescents and young adults 10–24 years	ICAP-funded YAFS with (1) training and mentorship for health care providers on care for adolescent/youth (2) one day per month at least about sexually and reproductive health, gynaecologic examination condoms, contraception (3) groups and education programme	Pre ART: – we observed decrease in LTFU between the pre- and post-YAFS but the difference was no significant *p* = 0.15 – no difference in LTFU between YAFS and non-YAFS facilities in pre YAFS period (*p* = 0.08) and no significant difference post (*p* = 0.87) YAFS period. Post ART: – no significant difference in LTFU in the before and after YAFS *p* = 0.19 – no significant difference in LTFU between YAFS and non-YAFS facilities in pre (*p* = 0.73) or post (*p* = 0.77) YAFS period – for health facilities that did not have YAFS, LTFU observed in the after period was significantly higher than the before period *p* = 0.04 – Data from the YAFS were evaluated immediately after implementation of the programme – may be a difference in the impact of YAFS on retention for adolescent compared with older youth – other factors may also contribute to the retention of this group
Nyabigambo A, Value Health 2014 [46]	Kampala, Uganda	Cross-sectional analysis looking at the association between adherence to clinic visits (attended all or missed 1 or more) and general wellbeing (measured with General Well Being Schedule, 18 point scale)	379 youth 15–24 years living with HIV who completed the general wellbeing schedule in 2012	Care in “HIV transition clinic” within infectious disease institute	Mean age 22 years, mean CD4 + 402.3, 60.9% currently on ART, 32.4% attended all clinic visits, no relationship between reported wellbeing and attending all clinical visits (compared to missing at least one visit)
Okoboi S, AIDS 2016 [50]	Uganda (TASO clinics)	Retrospective cohort analysis to examine retention of adolescents after ART and clinical factors associated with non-retention after ART	617 adolescents 10–19 years starting ART 2006–2011 at 6 TASO sites	Not specified	Overall retention: 90% at 12 months, 83% at 24 months and 76% at 36 months and 70% at 48 months Non-retention at 12-month associated: Clinic-based vs. community-based ART delivery (AHR 2.58, 95%CI 1.26–5.29) CD4 > 100 at ART initiation (AHR 1.38, 95%CI 1.01–1.90) 15–19 years (vs. 10–14) at ART initiation (AHR 1.88, 95%CI 1.01–3.48)
Vu, L AIDS 2016 [51]	Uganda	Evaluation of outcomes from Link Up intervention including self-efficacy for condom and contraceptive use, knowledge of HIV, condom use (last sex), HIV status disclosure, ART uptake and adherence, STI testing uptake, contraceptive prevalence	473 youth living with HIV 15–24 years who were members of Link Up peer support groups; 350 participated in follow-up survey	Link Up project funded by Alliance HIV: peer-led intervention through youth living with HIV peer support groups, counselling, HIV and reproductive health services and referral to ART and youth-oriented facilities	70% of participants were females; at the second interview there were significant increases in: Self-efficacy (AOR 1.8, 95%CI 1.3–2.6); Comprehensive HIV knowledge (AOR 1.8, 95%CI 1.3–2.6) HIV disclosure (AOR 1.6, 95%CI 1.01–2.6) Condom use at last sex (AOR 1.7, 95%CI 1.2–2.5) STI services uptake (AOR 2.1, 95%CI 1.5–2.9) ART uptake (AOR:2.5, 95%CI 1.6–4.0) ART adherence (AOR:2.5, 95%CI 1.3–4.9) CD4 testing (AOR:2.4, 95%CI 1.5–3.6) Use of contraceptives (AOR:1.7, 95%CI 1.1–2.7)

Further studies are needed to determine the factors that facilitate successful delivery of care for HIV-infected adolescents as they prepare to transition into adults care [48]. In a retrospective study comparing pre and post-ART retention before and after the implementation of youth-friendly services in Nyanza, Kenya, results suggest that offering a basic set of youth-friendly services, including dedicated clinics and support groups targeted to adolescents and youth, may not be adequate to surmount the retention barriers faced by adolescents and young adults [49]. Other factors such as socio-economic factors (food supplementation, schooling, employment and enhanced social support) or clinical (adherence) factors may also contribute to retention of this group and should be further understood [49]. There is a need to examine community-based approaches to reach and retain adolescents and youth to support treatment adherence, and to identify sub-groups that are at highest risk of dropping out for targeted care and support [50].

## Discussion

The objective of this review was to identify specific needs and challenges of HIV-infected adolescents and youth as they transition to adult HIV care in sub-Saharan Africa, and to describe existing models of care for transition and outcomes. We found that data on the adolescent transition of care and outcomes among HIV-infected youth after transition are still very limited in 2016. In part, this reflects the fact that transition of care is a relatively new concept and has only recently become an important component of care in sub-Saharan Africa; until the widespread availability of ART, most HIV-infected children died before reaching adolescence. Since 2004, the rollout of ART has allowed perinatally HIV-infected children to survive, leading to a larger population of these youth who have grown up with the infection, whose numbers are combined with those who were infected later in life [52].

While perinatally infected children are surviving longer, adolescents and young adults living with HIV in Africa represent a highly vulnerable and rapidly expanding patient group with unique developmental, psychosocial and comorbidity issues [4]. Managing the process of transitioning adolescents and youth from paediatric to adult care is particularly challenging in many resource-limited settings because healthcare systems are not adapted to their specific needs. In addition, collecting data on transition outcomes involves linking data sources from different points of care in clinical settings and requires longitudinal data. Despite these obstacles, it is critical to document transition outcomes and evaluate approaches in order to identify models that contribute to optimal patient outcomes.

In our review of available information on efforts to support transition, human resources and institutional challenges were highlighted as major barriers to adolescent transition, specifically the lack of training for staff to provide adolescent-friendly services [29,30]. These structural barriers are of critical concern and require comprehensive policy support and investments from national governments to train local healthcare providers. Stigma was also identified as a key barrier to transition in five studies [22,31–35]. Stigma, along with perceived lack of acceptance by communities causes adolescents and young adults to not disclose their status. A study in adults in Ethiopia reported that fear of stigma prevented patients from seeking emotional and instrumental support which could have improved their retention in care and clinical outcomes [53]. HIV disclosure places greater demands on healthcare providers who are already overworked. Furthermore, it causes avoidance of treatment centres and reluctance to attend support groups or utilize mobile Health. Increasing acceptance of adolescents and young adults in communities should be a focus area for interventions to support and improve outcomes in adolescent transition to adult care.

Owing to the paucity of data on HIV-infected adolescent’s transition to adult care in sub-Saharan Africa, we were unable to understand the impact of age at transition on outcomes for patients. The needs of adolescents during transition may also be different depending on the prior duration of ART (newly diagnosed versus established on treatment), and between perinatally versus behaviourally infected adolescents and youth. Data from sub-Saharan Africa are insufficient to answer these questions and should be further explored.

Our review included descriptions of the different models for adolescent transition that have been reported in the literature. Few studies have reported on models of care aimed at improving both pre and post ART retention. Support groups and greater involvement of adolescent healthcare providers have been suggested by healthcare providers themselves to facilitate transition [22,30]. Teen clinics have also been found in a small number of studies to improve retention compared to the standard model of care which is the most prevalent [48,50]. Although these youth-focused health services may improve their retention in HIV care, it is difficult to generalize study results due to their limited numbers, sample sizes, and short follow-up. In models where the same generalist healthcare workers may care for all age-groups, as in many community health centres, continuity is more naturally facilitated. However, additional training and sensitization for healthcare workers on adolescent issues should be provided. Comprehensive approaches to transition that assist healthcare providers, children adolescent and families can support smooth and successful transition into adult care setting [10]. Finally, it is important to note that these youth-friendly models are mainly funded by NGOs, international aid agencies, or through research efforts raising the question of how to sustain these efforts through health systems strengthening.

Rigorous evaluation of transition processes and specialized programmes in routine care settings is challenging as it requires monitoring of longitudinal outcomes data linked from different clinical points of care, and may require tracking different patient identifiers over time (e.g. with name changes) across multiple data sources. Improving longitudinal data gathering, standardization, analysis, and reporting systems specific to adolescent transition is essential to understanding and improving health outcomes.

Emerging data on outcomes after transition among HIV-infected youth are concerning as most studies report low retention and high mortality rates among adolescents and young adults post-transition. A recent study from the US HIV Research Network found that 19.8% of transitioned 21 year-olds were lost-to-follow-up after their 22nd birthday, and perinatally infected youth were less likely to have virologic suppression compared to behaviourally infected youth [54]. In a study conducted among perinatally infected adolescent and youth in the UK, 16–20-year olds treated in adult care were found to have significantly higher mortality compared to 13–15-year olds treated in paediatric care (rate ratio 2.7; 95% CI 0.6–12.2) [17]. A recent study conducted in Canada reported that a quarter of transitioned patients were no longer engaged in care [55]. Although these data are from high-income settings, we may expect similar findings in low-resource settings where report of transition outcomes are less common [48,50]. Descriptions of ongoing research efforts to capture transition outcomes in African settings and how they differ from high- and middle-income settings are necessary in order to identify key steps in the HIV cascade for interventions. Comprehensive care transition programme must be guided by research on health outcomes.

Several feasible recommendations have been identified including forming support groups and greater involvement of adolescent healthcare providers to facilitate the transition [30]. However, one size does probably not fit all when it comes to optimizing adolescent HIV care, and these approaches should be adapted to the health systems which themselves need to first acknowledge the need for supporting adolescent transition programmes. Interventions will need to take into account the specific needs of the adolescent and youth population to prevent morbidity and mortality during this vulnerable period of development and changes in health care.

## Conclusions

There are limited data on transition outcomes among HIV-infected adolescents and young adults in sub-Saharan Africa. More research in the management of adolescents is urgently needed, including documenting and comparing different models of transition. While evidence remains outstanding, it is likely that retaining adolescents and youth in care from the time of HIV diagnosis will require the implementation of a continuum of specific youth-friendly interventions including psychosocial support and clinical management, dedicated clinical days and spaces, and peer support. Another challenge will be to design and measure the impact of these interventions within these overburdened settings.
